# Redox active pyridine-3,5-di-carboxylate- and 1,2,3,4-cyclopentane tetra-carboxylate-based cobalt metal–organic frameworks for hybrid supercapacitors

**DOI:** 10.1039/d3ra03889k

**Published:** 2023-07-28

**Authors:** Muhammad Zahir Iqbal, Misbah Shaheen, Asma Khizar, Sikandar Aftab, Zubair Ahmad, Ahmed M. Tawfeek, Shahzad Sharif

**Affiliations:** a Nanotechnology Research Laboratory, Faculty of Engineering Sciences, Ghulam Ishaq Khan Institute of Engineering Sciences and Technology Topi 23640 Khyber Pakhtunkhwa Pakistan zahir@giki.edu.pk; b ZENTECH Research Laboratory, Faculty of Engineering Sciences, Ghulam Ishaq Khan Institute of Engineering Sciences and Technology Topi 23640 Khyber Pakhtunkhwa Pakistan; c Department of Intelligent Mechatronics Engineering, Sejong University 209 Neungdong-ro Gwangjin-gu Seoul 05006 South Korea aftab@sejong.ac.kr; d School of Chemical Engineering, Yeungnam University 280 Daehak-ro Gyeongsan Gyeongbuk 38541 Republic of Korea zubair7157@yu.ac.kr; e Department of Chemistry, College of Science, King Saud University Riyadh 11451 Saudi Arabia; f Department of Chemistry, Government College University Lahore Pakistan

## Abstract

In the pursuit of developing superior energy storage devices, an integrated approach has been advocated to harness the desirable features of both batteries and supercapacitors, particularly their high energy density, and high-power density. Consequently, the emergence of hybrid supercapacitors has become a subject of increasing interest, as they offer the potential to merge the complementary attributes of these two technologies into a single device, thereby surpassing the limitations of conventional energy storage systems. In this context the Metal–Organic Frameworks (MOFs), consisting of metal centers and organic linkers, have emerged as highly trending materials for energy storage by virtue of their high porosity. Here, we investigate the electrochemical performance of cobalt-pyridine-3,5-di-carboxylate-MOF (Co-PDC-MOF) and cobalt-1,2,3,4-cyclopentane tetra-carboxylate-MOF (Co-CPTC-MOF). In the setup involving the analysis of Co-PDC-MOF and Co-CPTC-MOF materials, a configuration comprising three electrodes was utilized. Drawing upon the promising initial properties of CPTC, a battery device was fabricated, comprising Co-CPTC-MOF, and activated carbon (AC) electrodes. Retaining a reversible capacity of 97% the device showcased impressive energy and power density of 20.7 W h g^−1^ and 2608.5 W kg^−1^, respectively. Dunn's model was employed, to gain deeper insights into the capacitive and diffusive contributions of the device.

## Abstract 2

In the pursuit of developing superior energy storage devices, an integrated approach has been advocated to harness the desirable features of both batteries and supercapacitors, particularly their high energy density and high-power density.

## Graphical abstract

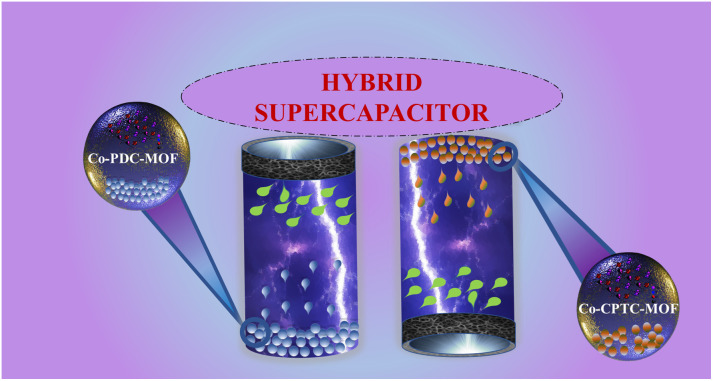

## 1. Introduction

A greener future without environmental and human cost is the key to sustainable life of mankind on Earth and the continuous utilization of fossil fuel is what shatters this dream. Thus, there is a dire need to reserve energy for future use.^1^ That is where energy storage devices like batteries and supercapacitors (SCs) spring up with the ability to harvest the stored energy after billions of years. Redox reactions happening in the bulk of batteries is what makes them capable of energy storage while the SCs, by bridging the functionality gap between batteries and conventional capacitors, work by accumulating energy *via* the distribution of charged ions in the electrolyte on the electrode surfaces.^2^ The challenge arises when there is a need for both high energy and power density, as conventional batteries and supercapacitors fail to meet these demands when used individually. Dendrite formation, volatility, electrolyte leakage and flammability are some of the issues that limits the productivity of batteries.^3^ On the other hand, SCs' lower energy density is due to their limited surface area and strength of electric field and physical reactions restricted to the surface of the electrode.^4^ Thus, to overcome the lower power density of and energy density of SCs, researchers are continuously exploring various electrode materials. In spite of the fact that there have been significant improvements in the performance of batteries and SCs but none of them can comply with the modern technology demand and will not be able to replace fossil fuels in the foreseeable future.^5^ Thus, the idea of getting the hybrid device by corroborating the features of batteries and supercapacitor is prevailing now a days. Such device of energy storage is termed as hybrid Supercapacitor.^6^ Therefore, to inspect novel materials for hybrid supercapacitor with souped-up electrochemical performance and reaction kinetic is highly crucial. While oxides of transition metals, phosphides, sulfides, and phosphates have all been widely studied for their possible uses, the search for new materials continues.^7,8^ Anticipated revolutionary advancements in batteries and supercapacitors involve the development of entirely novel categories of active electrode materials that improve surface area, electrical conductivity, and redox activity. One such category of materials *i.e.*, metal–organic frameworks (MOFs), surpasses even the porous nature of activated carbons.^9^ MOFs were first synthesized by Yaghi *et al.* in 1995 and have since generated considerable interest due to their multiple functions, easy processability, lower production cost, and customizable structures, all of which contribute to their superior electrochemical performance.^10^ MOFs have surfaced as extremely adaptable substances finding widespread applications across multiple domains, including catalysis, sensing, gas adsorption and separation, drug delivery, molecular sieves, energy storage and beyond.^11,12^ The exceptional properties and capabilities of MOFs have garnered significant attention and have positioned them as a prime area of research in the realm of materials science.^13^ Organic ligands shine as the driving force behind the electrifying potential of MOFs in energy storage applications in this regard.^14,15^

Herein, pyridine-3,5-dicarboxylate (PDC) and 1,2,3,4-cyclopentane tetra carboxylate (CPTC) is used as linker on account of their flexible characteristics. 3,5-PDC is a bidentate ligand, which means that it can coordinate with a metal center using two of its oxygen atoms, forming two separate bonds with the metal center enables the formation of strong metal–ligand bonds and thus can be used for making MOFs with tunable pore sizes.^16^ Employing PDC in MOFs can also lead to the formation of materials with high surface areas and tunable pore sizes.^17^ On contrary Cyclopentane tetracarboxylic acid (CPTC) possesses multiple coordination modes due to its unique molecular structure, featuring four carboxylic groups that can undergo partial or complete deprotonation. This property enables CPTC to act as both an electron acceptor and a donor, facilitating the formation of hydrogen bonds with other molecules. Furthermore, the aromatic chain of CPTC provides multiple attachment sites for transition metal ions, offering diverse options for chemical modification and functionalization. The presence of aromatic rings in CPTC engenders a favorable characteristic for their applicability in MOFs, which is the enhancement of the crystal structure through interactions like π-stacking between these rings.^18,19^ On the contrary Zhu *et al.* have used Co-MOF in his work with convincing results that at a current density of 2 mA g^−1^, the Co-MOF demonstrated an impressive specific capacitance of 14.7 F cm ^−2^.^20^ In a study by Jiao *et al.*, mixed-metal MOFs were employed to achieve supercapattery applications. The unique properties exhibited by MOFs, as highlighted earlier, confer a distinct advantage in their application as advanced materials in the realm of energy storage.^21^ The aforementioned unique characteristics of MOFs give them an edge to be used as advanced materials for energy storage applications.

Our research endeavors culminate in the production of two unique MOFs featuring cobalt (Co), which were synthesized utilizing the hydrothermal method. Following the synthesis process, to gain a valuable insight into their potential applications we performed a comprehensive characterization of each MOF sample through X-ray diffraction (XRD) and scanning electron microscopy (SEM). To divulge electrochemical attributes of both the MOFs, three-electrode assembly is utilized and then the best performing MOF was adapted to fabricate a hybrid supercapacitor with AC. Compared to traditional materials, it has been established that Co-based MOFs offer significant potential in the future for further exploitation as an energy storage material.^22,23^

## 2. Experimental

### 2.1. Materials and methods

Source chemicals including cobalt(ii) nitrate, pyridine 3,5-dicarboxylic acid, 1,2,3,4-cyclopentane tetracarboxylic acid were purchased from Sigma-Aldrich. All the precursors were of 99.99% purity and utilized without additional processing.

### 2.2. Synthesis of pyridine 3,5-dicarboxylate (Co-PDC)/cobalt-1,2,3,4-cyclopentane tetra-carboxylate (Co-CPTC)-MOF

**Fig. 1:**
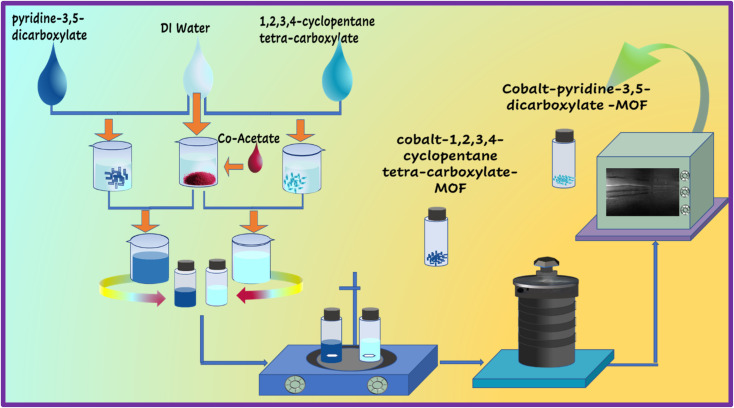
Schematic of hydrothermal process used for MOFs synthesis.

The hydrothermal approach was used to create Co-PDC-and-CPTC-MOF, as reported in prior studies.^24^ A solution was prepared by mixing deionized water (DI water) and 0.5 mM cobalt(ii) nitrate. In a separate step, a solution containing 0.3 mM pyridine 3,5-dicarboxylic acid for Co-PDC-MOF and 1,2,3,4-cyclopentane tetracarboxylic acid for Co-CPTC-MOF was also made using DI water, followed by vigorous magnetic stirring with a previous solution. After the solution was prepared, it was heated in a Teflon-lined autoclave at a temperature of 130 °C for 48 hours. After obtaining Co-PDC-MOF crystals, they were cooled to room temperature, centrifuged, and cleaned with ethanol, acetone, and DI water before being dried to remove impurities.^25^ The schematic of the above-mentioned method is elaborated in Fig. 1.

## 3. Characterization techniques

### 3.1. Electrode fabrication

In this study, a working electrode was created by depositing a MOF onto a nickel foam (NF) substrate. Prior to deposition, the NF was subjected to a cleaning process involving sequential washes with hydrochloric acid, acetone, ethanol, and methanol. The material deposited consisted of 80 wt% active material, 10 wt% polyvinylidene fluoride (PVDF) binder, and 15 wt% acetylene black, which were dispersed in *N*-methyl-2-pyrrolidone (NMP) solvent. To ensure a uniform coating of the nickel foam surface, all the aforementioned materials were continuously stirred magnetically for 8 hours to create a homogeneous slurry. The same procedure was followed for the deposition of both Co-PDC-MOF and Co-CPTC-MOF materials.

### 3.2. Electrochemical measurements

Cyclic voltammetry (CV), galvanostatic charge–discharge (GCD), and electrochemical impedance spectroscopy (EIS) were used to examine the electrochemical properties of Co-PDC-MOF and Co-CPTC-MOF. Assembly of three electrodes was established for this purpose, all performed in a potassium hydroxide solution with a concentration of 1 M. The specific capacities of the electrode material were determined at different potential sweep rates in CV using the equation below:1
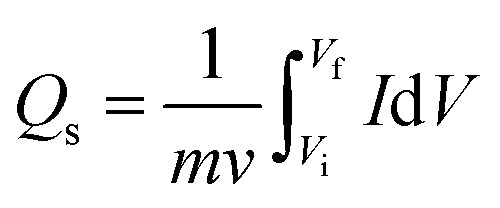


This equation provides the relation of specific capacity, where ‘*m*’ represents the active mass of the synthesized nanomaterial, “*V*” denotes the potential sweep rate, and the integral reveals the area under the CV profile. Initially, CV was executed at scan rates ranging from 3–50 mV s^−1^. GCD was also conducted at diverse current densities. The electrode was charged and discharged 1000 times in order to evaluate the cyclic stability. Moreover, EIS experiments within a frequency range of 0.01–100 kHz at 5 mV AC voltage were used to measure the conductivity of the electrodes *via* GCD, eqn (2) was used to calculate the specific capacities (*Q*_s_) of the two MOFs:^26^2
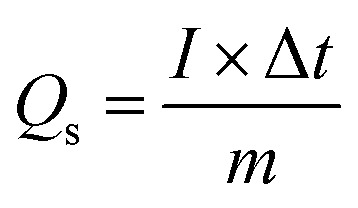
Here discharge current density (*I*/*m*) expressed in milliamperes per gram (mA g^−1^), the discharging time (Δ*t*) denoted in seconds (s), and *Q*_s_ measured in Coulombs per gram (C g^−1^).^27^

## 4. Results and discussion

### 4.1. Structural and morphological analysis

**Fig. 2:**
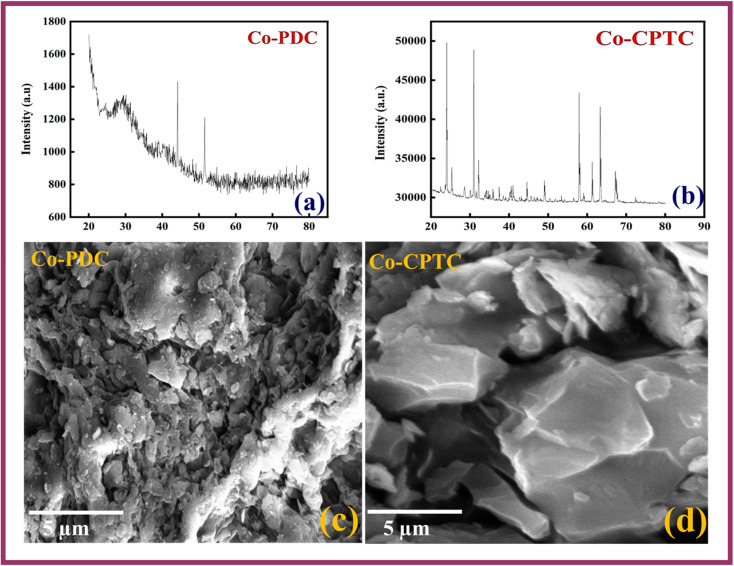
XRD of (a) Co-PDC MOF (b) Co-CPTC MOF and SEM of (c) Co-PDC MOF (d) Co-CPTC MOF.

XRD technique has been used to get insight of the crystal structure of synthesized MOFs as shown in Fig. 2(a and b). Clear, refined, and more peaks are observed in the case of Co-CPTC (CCDC 793234) while relatively amorphous nature is observed in case of Co-PDC (CCDC 296674). The crystalline nature of Co-CPTC is further confirmed through the SEM micrographs too as shown in Fig. 2(c and d).

### 4.2. Half-cell electrochemical analysis

**Fig. 3:**
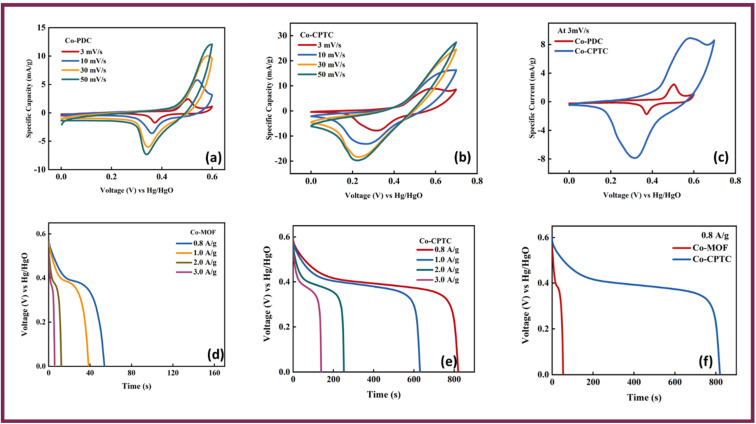
CV profiles of (a) Co-PDC MOF, (b) Co-CPTC MOF. (c) CV comparison at 3 mV s^−1^ for both MOFs GCD profiles of (d) Co-PDC MOF (e) Co-CPTC MOF, (f) GCD comparison at 0.8 A g^−1^ for both the MOFs.

In order to unravel the electrochemical characteristics inherent in Co-PDC-MOF and Co-CPTC-MOF, respectively, we conducted the CVs at different scan rates (3, 10, 30, and 50 mV s^−1^) to elucidate the charge storage mechanism as depicted in Fig. 3(a and b). The CV result reveals that battery-like characteristics is observed in both Co-PDC-MOF and Co-CPTC-MOF which is evident by pronounced redox active peaks. The emergence of these peaks can be attributed to the OH^−1^ ions found in the electrolyte interacting with electrode, leading to oxidation and reduction reactions. Overall, our results suggest that Co-CPTC exhibits better reaction kinetics owing to its peak along with large area under the curve, highlighting its potential as a promising candidate for electrochemical applications. These oxidation–reduction peak currents are crucial for understanding the electrochemical behavior at the electrode–electrolyte interface, as they reflect the reversible redox processes occurring during the charge–discharge cycles. The symmetry of curves indicates that redox reactions are reversible. In Fig. 3(c), CVs of both the MOFs are shown at scan rate of 3 mV s^−1^ for the comparative analysis.

Furthermore, GCD was performed at current densities of 0.4, 0.8, 1.0, 1.6, 2.0, and 3.0 A g^−1^ of Co-PDC-MOF and Co-CPTC-MOF, and the discharge time were plotted in Fig. 3(d and e). While an increase in current density resulted in a decrease in discharge time, the discharge plateaus observed in the GCD curves remained relatively constant, demonstrating the materials stability even at higher current densities. At the electrode surface, the reaction kinetics may be affected by insufficient time for proper intercalation of OH^−^ ions, resulting in inadequate filling of the sites where ions undergo redox reactions and adsorbed. The phenomenon can lead to a decline in discharge time, indicating the importance of efficient ion intercalation for optimal device performance.^28^ The linear zone at the beginning and end of the discharge curve is caused by the creation of the diffusive double layer at the interface of electrode–electrolyte. During this process, the electrostatic interaction between charges of the electrode and electrolyte ions gives rise to a potential difference that can slow down the charge transfer process. Yet, despite this delay, the GCD curves reveal that a significant portion of the curves remain non-linear, provide compelling evidence of redox activity during the process.^29^ In comparison to Co-PDC-MOF, the GCD curves of Co-CPTC-MOF demonstrated a considerably larger discharge plateau which reveals its large discharging time and better performance as battery grade material (Fig. 3f).

**Fig. 4:**
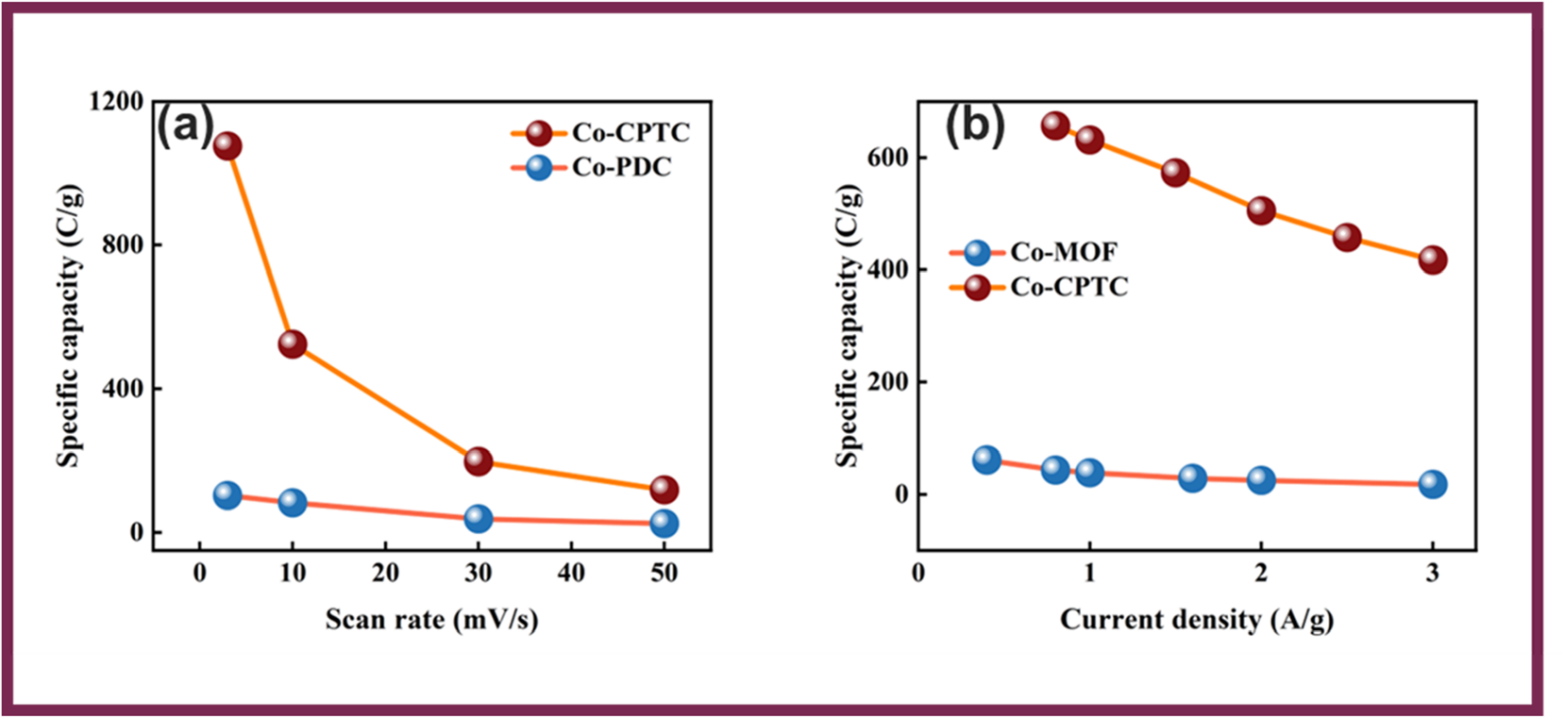
(a) Specific capacities of both MOFs *vs.* scan rate (b) specific capacities of both MOFs *vs.* current density.

Fig. 4(a) showcases the specific capacity (*Q*_s_) of Co-PDC-MOF and for Co-CPTC-MOF against varying scan rates from the CVs and Fig. 4(b) shows current densities extracted from the GCD curves, revealing maximum achieved *Q*_s_ of (CV = 133 C g^−1^, GCD = 83 C g^−1^) and (CV = 1095 C g^−1^, GCD = 679 C g^−1^) for Co-PDC-MOF and Co-CPTC-MOF, respectively. The battery-grade performance of Co-CPTC-MOF shows relatively better performance as compared to Co-PDC-MOF making it a promising electrode material for hybrid supercapacitor applications.^30^ The structure of the ligand in Co-CPTC-MOF could result in a more complex and flexible coordination environment for the metal ion compared to the linear ligand in Co-PDC-MOF, which could results in higher specific capacity in case of Co-CPTC-MOF.^31^

**Fig. 5:**
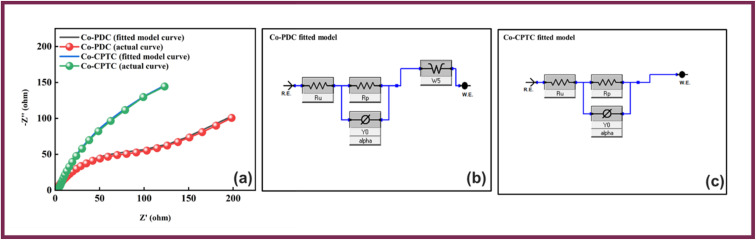
(a) EIS results of Co-PDC MOF and Co-CPTC MOF along with the fitted model curves (b and c) fitted models for EIS of Co-PDC and Co-CPTC.

The conductive and interfacial properties of the electrodes were analyzed using EIS in the frequency range of 0.1 Hz to 100 kHz. Fig. 5(a), shows the Nyquist diagrams along with the fitted model curves of Co-CPTC-MOF and Co-PDC-MOF where Co-CPTC-MOF exhibits a non-semicircular plot while Co-PDC-MOF shows a slight deviation from a semicircular shape, which could be due to irregular electrochemical behavior or the electrode surface roughness. The equivalent series resistance (ESR), which exists between the electrolyte and electrode, was derived by identifying the point of intersection on the high-frequency abscissa. The ESR values of Co-CPTC-MOF and Co-PDC-MOF were found to be 1.3 Ω and 1.6 Ω, respectively, indicating the relatively conductive nature of the material.^32^ The fitted models for EIS of Co-PDC and Co-CPTC are shown in Fig. 5(b and c) respectively.

### 4.3. Hybrid supercapacitor assembly

**Fig. 6:**
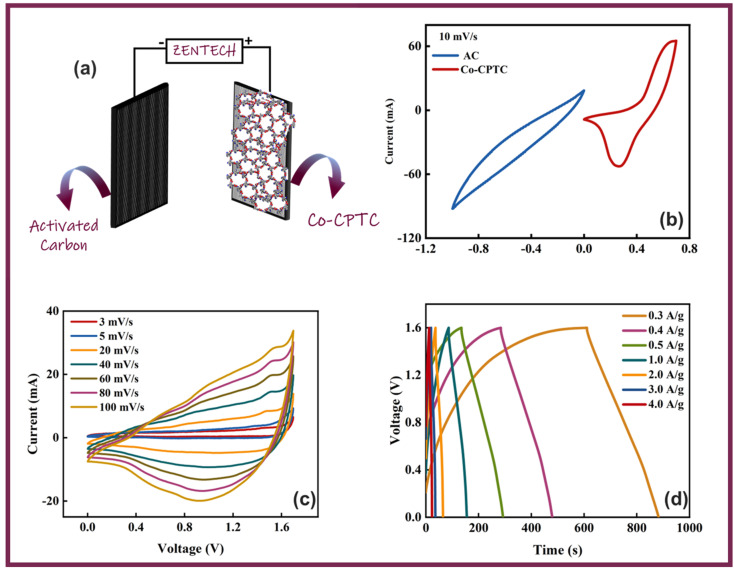
(a) Schematic illustration of fabricated Co-CPTC MOF//AC device (b) CV comparison at 10 mV s^−1^ for Co-CPTC MOF and AC, (c) CV curves of Co-CPTC MOF//AC (d) GCD curves of Co-CPTC MOF//AC.

**Fig. 7:**
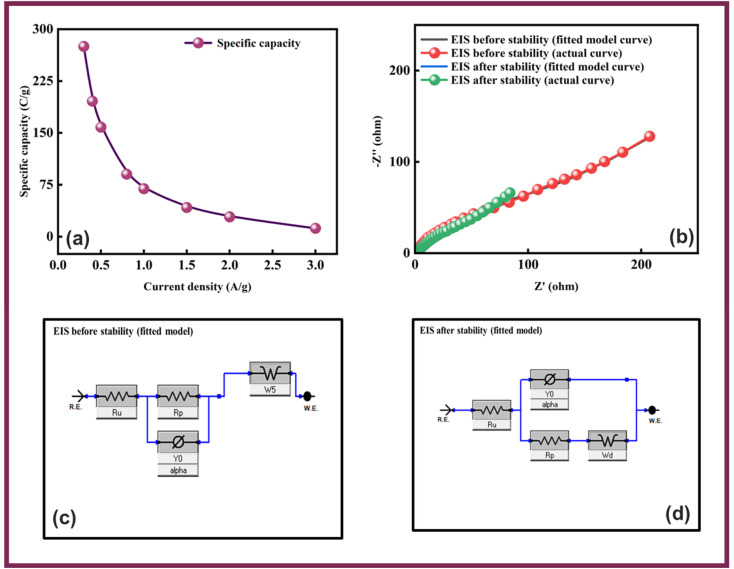
(a) Specific capacities *vs.* current density of fabricated device (b) EIS along with the fitted model curves of Co-CPTC MOF//AC device before and after stability (c and d) fitted models for EIS of Co-CPTC before and after stability.

**Fig. 8:**
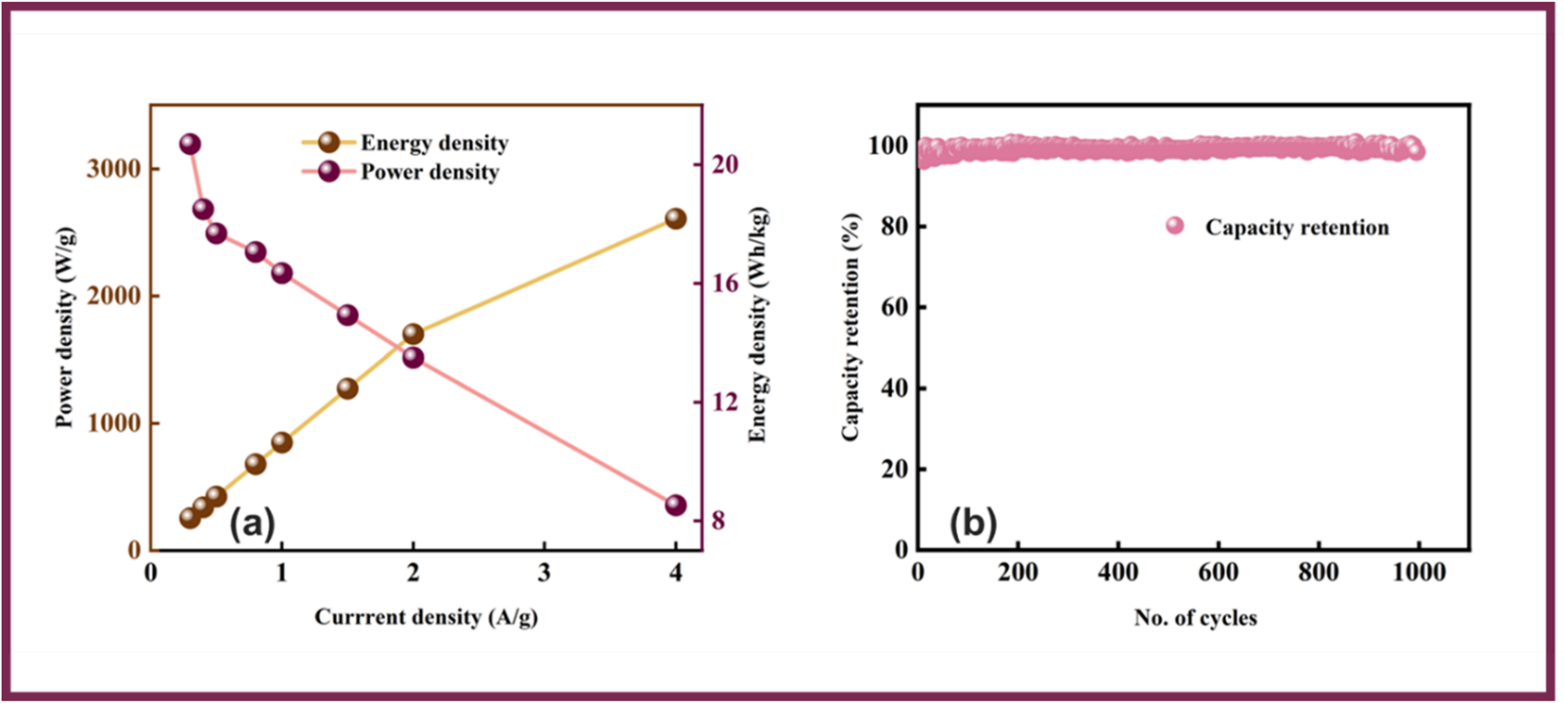
(a) Energy and power density of AC//Co-CPTC MOF device (b) capacity retention of Co-CPTC MOF//AC device.

**Fig. 9:**
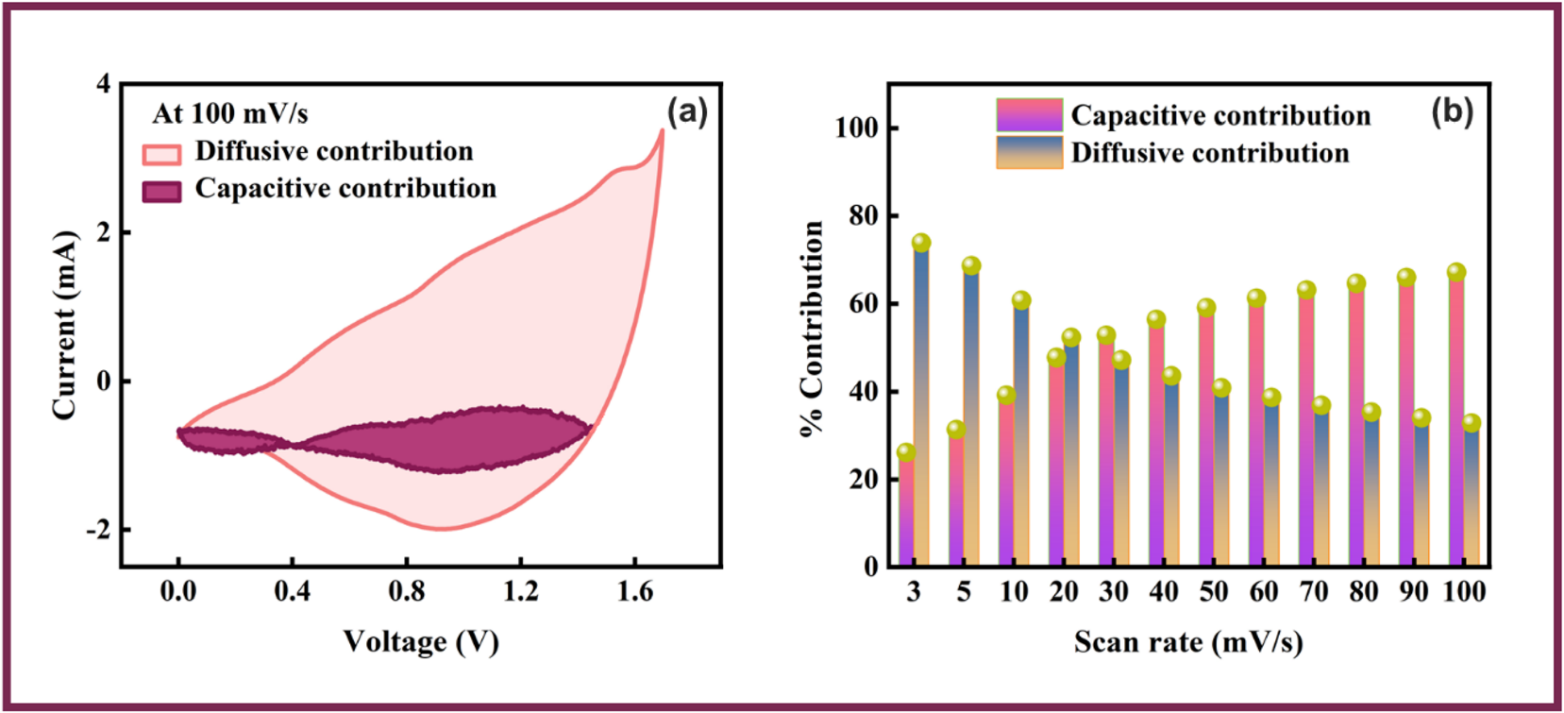
Fitting of capacitive and diffusive part at (a) 100 mV s^−1^. (b) Percentage capacitive and diffusive contributions.

Owing to the impressive capabilities of Co-CPTC-MOF, we make a real device with it by employing activated carbon (AC) as the negative electrode and this MOF as positive electrode, as shown in Fig. 6(a). In order to meticulously evaluate the fabricated device (Co-CPTC-MOF//AC), two electrode setup is employed. Fig. 6(b) displaying the CV of both the AC as capacitive electrode with area under the curve and Co-CPTC-MOF as battery grade electrode at scan rate of 10 mV s^−1^. By utilizing this approach, it becomes possible to discern the electrochemical contributions attributed to electrostatic capacitance and battery-like behavior. The rectangular shape in the CV curve is indicative of the electrode's charge storage capacity *via* non-faradaic processes while Co-CPTC-MOF the Faradaic nature. The CV of the device was captured at seven different potential sweeps depicted in Fig. 6(c). The CV curves of our hybrid device, which utilizes both capacitive (AC) and battery-grade (Co-CPTC-MOF) electrodes, exhibit a combination of redox peaks and area under the curve, clearly demonstrating the hybrid nature of the device. The consistency in the shape of the voltammograms, even at increased potential scans, indicates the device's stability and reliability. To quickly assess the device's practical applicability, voltage graphs with charge discharging time at seven distinct current densities are presented in Fig. 6(d) depicting the device's discharge at varying current densities. The slight non-linearity observed in the curves indicates the coexistence of both faradaic and non-faradaic reactions, implying a combination of capacitive and diffusive elements. The specific capacity of the Co-CPTC-MOF//AC device was quantified using the GCD method, yielding 283.4 C g^−1^ at 0.3 A g^−1^. The *Q*_s_*vs.* various current densities trend is plotted in Fig. 7(a). The device's capacity decreased with increasing current densities, likely due to inadequate interaction time of ions with the electrode. Conductivity is a crucial aspect of the devised hybrid supercapacitor, and the device's conductive properties were assessed using EIS before and after stability test as shown in Fig. 7(b) along with the fitted model curves. The EIS results revealed a low equivalent series resistance (ESR) value of 0.4 Ω and 0.1 Ω before and after stability, respectively. The fitted models for EIS of Co-CPTC before and after stability are shown in Fig. 7(c and d) respectively. Using the specific capacity obtained from GCD, the energy density (*E*_s_) and power density (*P*_s_) were calculated and plotted in Fig. 8(a). The following equations were employed to determine *E*_s_ and *P*_s_, respectively:3
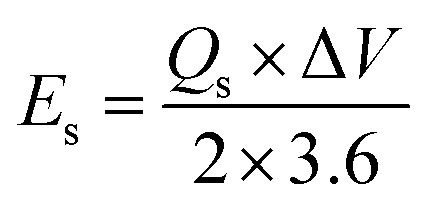
4
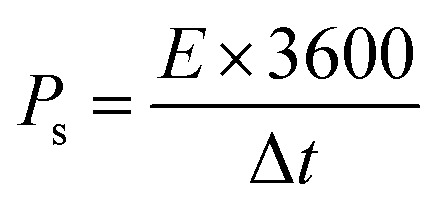
In above equations, *Q*_s_ presents the specific capacity, Δ*V* demonstrate the optimal potential window and Δ*t* denotes the discharging time. The Co-CPTC-MOF//AC device demonstrated impressive power and energy densities, delivering *E*_s_ of 20.7 W h kg^−1^ at *P*_s_ of 2608.5 W kg^−1^ when operated at a current density of 0.3 A g^−1^. The device's stability was assessed by subjecting it to 1000 GCD cycles, resulting in 97% capacity retention, demonstrating its exceptional potential as displayed in Fig. 8(b). The hybrid nature of the device was further validated through simulation using Dunn's model, where the capacitive and diffusive contributions were analyzed at raised scanning frequencies. This was evident from the fitting of the experimental results of CV at 100 mV s^−1^ as shown in Fig. 9(a), where the capacitive portion was observed to be more pronounced. Fig. 9(b) further substantiated this observation, exhibiting the percentages of capacitive and diffusive contributions. When scanning rates were increased, the ions were allotted a restricted time to execute redox reactions, which resulted in a more significant capacitive response.

The Co-CPTC-MOF//AC device displayed better electrochemical performance due to the synergistic effects resulting from the utilization of two electrodes of different natures. The power and stability of the device was provided by the AC electrode, while the Co-CPTC-MOF electrode compensated for the low energy density of the AC electrode.

## 5. Conclusion

Our study investigates the configuration of employing MOFs such as Co-PDC-and-CPTC-MOF for hybrid supercapacitor applications. Initial characterization is performed through XRD and SEM followed by half-cell electrochemical characterization. The Co-CPTC-MOF was found to have a higher specific capacity compared to the Co-PDC-MOF in half-cell measurements. Considering the relatively better performance of Co-CPTC-MOF than Co-PDC, it was employed as the operational positive electrode along with AC as negative electrode in a hybrid supercapacitor device, yielding a specific capacity of 283.4 C g^−1^, *E*_s_ (20.7 W h kg^−1^) and *P*_s_ (2608.5 W kg^−1^), respectively. Further, the device exhibits good cyclic stability, highlighting its potential in energy storage devices. By utilizing the Dunn's model, an evaluation was conducted to evaluate the capacitive and diffusive contributions of the device. These findings showcase the potential and contribution of each electrode in the device. Altogether, this study offers valuable perspectives on the diverse characteristics of MOFs and their viability as electrode materials in energy storage systems.

## Conflicts of interest

There are no conflicts to declare.

## Supplementary Material

## References

[cit1] Connolly B. M., Mehta J. P., Moghadam P. Z., Wheatley A. E., Fairen-Jimenez D. (2018). Curr. Opin. Green Sustainable Chem..

[cit2] Dubal D. P., Ayyad O., Ruiz V., Gomez-Romero P. (2015). Chem. Soc. Rev..

[cit3] Kim K. J., Balaish M., Wadaguchi M., Kong L., Rupp J. L. M. (2021). Adv. Energy Mater..

[cit4] Vinodh R., Muralee Gopi C. V. V., Yang Z., Deviprasath C., Atchudan R., Raman V., Yi M., Kim H.-J. (2020). J. Energy Storage.

[cit5] Zhang H., Xu B., Xiao Z., Mei H., Zhang L., Han Y., Sun D. (2018). CrystEngComm.

[cit6] Iqbal M. Z., Aziz U. (2022). J. Energy Storage.

[cit7] Theerthagiri J., Murthy A. P., Lee S. J., Karuppasamy K., Arumugam S. R., Yu Y., Hanafiah M. M., Kim H.-S., Mittal V., Choi M. Y. (2021). Ceram. Int..

[cit8] Iqbal M. Z., Khan J. (2021). Electrochim. Acta.

[cit9] Zahir Iqbal M., Amjad N., Waqas Khan M. (2022). ChemElectroChem.

[cit10] Yaghi O., Li H. (1995). J. Am. Chem. Soc..

[cit11] Yang D., Gates B. C. (2019). ACS Catal..

[cit12] Kumar P., Deep A., Kim K.-H. (2015). TrAC, Trends Anal. Chem..

[cit13] Mehtab T., Yasin G., Arif M., Shakeel M., Korai R. M., Nadeem M., Muhammad N., Lu X. (2019). J. Energy Storage.

[cit14] Yaghi O. M., Li G., Li H. (1995). Nature.

[cit15] Lin Z.-J., Lü J., Hong M., Cao R. (2014). Chem. Soc. Rev..

[cit16] Cui J.-Z., Zhang H., Zhang D., Wang H.-T., Gao H.-L. (2006). Acta Crystallogr., Sect. E: Struct. Rep. Online.

[cit17] Iqbal M. Z., Khan M. W., Shaheen M., Siddique S., Aftab S., Wabaidur S. M., Sharif S. (2023). Mater. Today Sustainability.

[cit18] Yao R.-X., Hao Z.-M., Guo C.-H., Zhang X.-M. (2010). CrystEngComm.

[cit19] Huang L.-F., Ji C.-C., Lu Z.-Z., Yao X.-Q., Hu J.-S., Zheng H.-G. (2011). Dalton Trans..

[cit20] Zhu G., Wen H., Ma M., Wang W., Yang L., Wang L., Shi X., Cheng X., Sun X., Yao Y. (2018). Chem. Commun..

[cit21] Peng Y., Zhou T., Ma J., Bai Y., Cao S., Pang H. (2022). Adv. Colloid Interface Sci..

[cit22] Wang K., Li Q., Ren Z., Li C., Chu Y., Wang Z., Zhang M., Wu H., Zhang Q. (2020). Small.

[cit23] Wang K., Guo Y., Zhang Q. (2022). Small Struct..

[cit24] Hong J., Park S.-J., Kim S. (2019). Electrochim. Acta.

[cit25] Qi Y., Luo F., Che Y., Zheng J. (2008). Cryst. Growth Des..

[cit26] Acharya D., Pathak I., Dahal B., Lohani P. C., Bhattarai R. M., Muthurasu A., Kim T., Ko T. H., Chhetri K., Kim H. Y. (2023). Carbon.

[cit27] Iqbal M. Z., Shaheen M., Khan M. W., Siddique S., Aftab S., Wabaidur S. M., Iqbal M. J. (2023). RSC Adv..

[cit28] Jahromi S. P., Pandikumar A., Goh B. T., Lim Y. S., Basirun W. J., Lim H. N., Huang N. M. (2015). RSC Adv..

[cit29] Akinwolemiwa B., Peng C., Chen G. Z. (2015). J. Electrochem. Soc..

[cit30] Iqbal M. Z., Shaheen M., Siddique S., Aftab S., Wabaidur S. M. (2023). J. Energy Storage.

[cit31] Iqbal M. Z., Aziz U., Aftab S., Wabaidur S. M., Siddique S., Iqbal M. J. (2023). ChemistrySelect.

[cit32] Duraisamy N., Numan A., Fatin S. O., Ramesh K., Ramesh S. (2016). J. Colloid Interface Sci..

